# COVID-19 in Infants Less than 3 Months: Severe or Not Severe Disease?

**DOI:** 10.3390/v14102256

**Published:** 2022-10-14

**Authors:** Daniele Dona’, Carlotta Montagnani, Costanza Di Chiara, Elisabetta Venturini, Luisa Galli, Andrea Lo Vecchio, Marco Denina, Nicole Olivini, Eugenia Bruzzese, Andrea Campana, Roberta Giacchero, Filippo Salvini, Antonella Meini, Matteo Ponzoni, Sandra Trapani, Elena Rossi, Mary Haywood Lombardi, Raffaele Badolato, Luca Pierri, Giulia Pruccoli, Sara Rossin, Claudia Colomba, Salvatore Cazzato, Ilaria Pacati, Giangiacomo Nicolini, Luca Pierantoni, Sonia Bianchini, Andrzej Krzysztofiak, Silvia Garazzino, Carlo Giaquinto, Guido Castelli Gattinara, on behalf of The Italian SITIP-SIP Pediatric SARS-CoV-2 Infection Study Group

**Affiliations:** 1 Division of Pediatric Infectious Diseases, Department for Women’s and Children’s Health, University of Padua, 35128 Padua, Italy; 2 Pediatric Infectious Diseases Division, Anna Meyer Children’s University Hospital, 50139 Florence, Italy; 3 Department of Health Sciences, University of Florence, 50121 Florence, Italy; 4 Department of Translational Medical Sciences, University of Naples Federico II, 80138 Naples, Italy; 5 Department of Pediatrics, Infectious Diseases Unit, University of Turin, Regina Margherita Children’s Hospital, 10126 Turin, Italy; 6 Ospedale Pediatrico Bambino Gesù, UOC Pediatria Multispecialistica, Fiumicino, 00050 Rome, Italy; 7 UOC Pediatria-ASST Lodi, 26900 Lodi, Italy; 8 Department of Pediatrics, Niguarda Hospital, 20162 Milan, Italy; 9 Department of Experimental and Clinical Sciences, Pediatric Clinic, University of Brescia, 25121 Brescia, Italy; 10 Pediatric and Congenital Cardiac Surgery Unit, Department of Cardiac, Thoracic and Vascular Sciences, University of Padova, 35128 Padova, Italy; 11 Pediatric Emergency Department, Department for Woman and Child Health, University of Padua, 35128 Padua, Italy; 12 Department of Health Promotion, Mother and Child Care, Internal Medicine and Medical Specialties, Infectious Diseases Unit, University of Palermo, 90133 Palermo, Italy; 13 Paediatric Unit, Department of Mother and Child Health, Salesi Children’s Hospital, 60123 Ancona, Italy; 14 Paediatric Unit, Ospedale Bolognini ASST Bergamo Est, 24068 Seriate, Italy; 15 Pediatric Unit, San Martino Hospital, 32100 Belluno, Italy; 16 Medical and Surgical Science Department, S Orsola University Hospital, 40138 Bologna, Italy; 17 Pediatric Clinic, Department of Surgical and Biomedical Sciences, Università degli Studi di Perugia, 06132 Perugia, Italy; 18 Pediatric and Infectious Diseases Unit, Academic Department, Bambino Gesù Pediatric Hospital, 00146 Rome, Italy; 19 Universitarian-Hospital Department, Ospedale Bambino Gesù IRCCS, 00146 Rome, Italy

**Keywords:** SARS-CoV-2, COVID-19, neonates, infants

## Abstract

Compared to adults, severe or fatal COVID-19 disease is much less common in children. However, a higher risk for progression has been reported in infants. Different pediatric COVID-19 severity scores are reported in the literature. Methods: Subjects under 90 days of age admitted to 35 Italian institutions for COVID-19 were included. The severity of COVID-19 was scored as mild/moderate or severe/critical following the classification reported in the literature by Venturini, Dong, Kanburoglu, and Gale. To assess the diagnostic accuracy of each classification system, we stratified all enrolled patients developing a posteriori severity score based on clinical presentation and outcomes and then compared all different scores analyzed. Results: We included 216 infants below 90 days of age. The most common symptom was fever, followed by coryza, poor feeding, cough, and gastrointestinal manifestations. According to Venturini, Dong, Kanburoglu, and Gale’s severity scores, 18%, 6%, 4.2%, and 29.6% of infants presented with severe/critical disease, respectively. A correlation analysis between these four scores and the a posteriori severity score assigned to all enrolled subjects was performed, and a crescent strength of correlation from Gale (R = 0.355, *p* < 0.001) to Venturini (R = 0.425, *p* < 0.001), Dong (R = 0.734, *p* < 0.001), and Kanburoglu (R = 0.859, *p* < 0.001) was observed. Conclusions: The percentage of infants with severe COVID-19 varies widely according to the score systems. A unique clinical score should be designed for neonates and infants with COVID-19.

## 1. Introduction

The coronavirus disease 2019 (COVID-19) pandemic, caused by severe acute respiratory syndrome coronavirus 2 (SARS-CoV-2), continues to expand. From its identification in December 2019 to the time of writing, SARS-CoV-2 has caused COVID-19 in more than 500 million people worldwide and resulted in 6.3 million deaths [1].

COVID-19 clinical manifestations range from asymptomatic infection to severe pneumonia with acute respiratory distress syndrome [2,3]. Children are largely spared from a severe respiratory illness compared to adults; however, a higher risk for progression to severe COVID-19 has been reported in infants and children with underlying medical conditions [2,3,4,5,6,7,8,9].

Whereas comorbidities are recognized as risk factors for severe disease progression, data on age-related poor prognosis are still debated [10].

Among 2143 children reported by Dong et al., 90% ranged from asymptomatic to moderate COVID-19, with a higher prevalence of severe and critical cases equal to 3.0% in adolescents and 10.6% in children under 5 years old [7]. Moreover, in a prospective UK population-based cohort study, Gale et al. showed that neonatal SARS-CoV-2 infection led to severe disease in 42% of cases and highlighted that 33% of babies required respiratory support [11].

Conversely, two meta-analyses have contradicted these findings, reporting that SARS-CoV-2 infected neonates are usually asymptomatic or with a mild disease similar to older children [12,13]. Moreover, Gotzinger et al., in a retrospective multicenter study, observed that most neonates requiring intensive care had severe comorbidities comprising cardiac anomalies and prematurity. Overall, 31/35 neonates did not require respiratory support at any stage, and almost one in five remained entirely asymptomatic. The proportion of neonates requiring ventilatory support did not differ from that observed in older children [14].

Currently, scientific knowledge investigating the clinical features and outcomes of COVID-19 in neonates and infants is scarce and contradictory.

In the literature, different classifications have been used to score the severity of COVID-19 in neonates and children. Dong et al. classified patients who presented hypoxemia as severe, and those with any type of organ dysfunction as critical [7]. Gale et al. considered patients presenting with a combination of clinical symptoms, alteration of blood cell counts and C-reactive protein, and abnormalities of a chest X-ray as severe (including critical cases) [11]. Venturini et al. scored patients with respiratory distress or systemic symptoms (drowsiness, lethargy, seizures, dehydration) as severe, and patients with pediatric acute respiratory distress syndrome, sepsis-associated organ dysfunction, septic shock, or coma as critical [15]. Lastly, Kanburoglu et al. included the need for nasal continuous positive airway pressure in the severe group and considered critical patients as those needing mechanical ventilation or presenting with disseminated intravascular coagulopathy or multiple organ dysfunction [16].

Therefore, to better understand COVID-19 in young children, we describe the epidemiological, clinical, and outcome aspects of SARS-CoV-2 infection in neonates and infants less than 3 months old and compare the different COVID-19 severity scores reported in the literature for this pediatric population.

## 2. Materials and Methods

### 2.1. Study Design and Data Collection

The present study is a sub-analysis of the data collected from a multicenter pediatric network including 62 institutions across Italy [17].

All subjects under 90 days of age admitted with a laboratory-confirmed SARS-CoV-2 infection from 26 February 2020 to 4 February 2021 were included. Diagnosis of infection was established with at least one respiratory specimen positive for SARS-CoV-2 nucleic acid using a validated real-time reverse-transcriptase polymerase-chain-reaction (RT-PCR) assay performed at hospital admission.

Data were de-identified, recorded through a targeted registration form by each pediatrician involved, and transferred to a specifically designed database anonymously. Patient-based data included health status, previous medical and vaccination history, laboratory and microbiological reports, COVID-19-related diagnosis and clinical details, prescriptions, diagnostic procedures, hospital admissions, and outcome data. A follow-up of at least 2 weeks was required to outline the infection’s outcome. Data collection was allowed by at least one parent’s written consent for active participation in the study.

### 2.2. Evaluation of COVID-19 Outcomes and Severity Scores Assessment

The severity of COVID-19 was scored as mild/moderate or severe/critical following the classification reported in the literature by Dong, Gale, Venturini and Kanburoglu [7,11,14,15] (Table 1).

To assess the predictive value of the four scores, we stratified all enrolled patients a posteriori based on their needing for pharmacological, oxygen, and intensive support and on their outcomes as specified below, then compared them to each other.

A severity score was adopted to assess clinical outcomes of COVID-19 as follows:

- SCORE 1—clinical and/or radiological diagnosis of pneumonia with normal oxygen levels or mild specific involvement of a single organ/apparatus without specific pharmacological support, requiring hospitalization without ICU admission.
- SCORE 2—acute respiratory distress syndrome requiring low or high flow (HFNC) oxygen therapy without ICU admission, or moderate specific involvement of a single organ/apparatus with the need of specific pharmacological support, requiring hospitalization without ICU admission.
- SCORE 3—severe acute respiratory illness (SARI) (SpO2 < 92% associated with tachypnea and other signs of respiratory failure requiring non-invasive or invasive mechanical ventilation, or critical involvement of a single organ/apparatus with the need of specific pharmacological support with ICU admission.
- SCORE 4—Death

Patients who did not satisfy the 1, 2, 3, and 4 criteria were considered mild COVID-19 cases (score 0).

### 2.3. Statistical Analyses

Statistical analysis was performed using IBM SPSS Statistics 25.0 (IBM Corp. Armonk, NY, USA). Statistical significance was set at *p* < 0.05. All *p*-values were 2-tailed. In the descriptive analysis, categorical variables were expressed as percentages and continuous variables as median and interquartile range (IQR). Categorical variables were assessed in a RxC table with chi-squared or Fisher exact test as appropriate. The correlation between neonates’ clinical severity scores and the four scores mentioned above was measured using Pearson’s R Coefficient.

## 3. Results

### 3.1. Patient Characteristics

The present analysis included 216 infants below 90 days of age admitted to 35 hospitals. In total, 124 infants were male (57.4%). At diagnosis, the median age was 43 days (IQR 25–64), and 59/216 (27.3%) patients were newborns. Two infants were born from a positive mother and tested positive in the first 48 h of life. Overall, 138 (63.9%) infants had known contact with SARS-CoV-2; mothers resulted SARS-CoV-2 positive in 169/216 (78.2%) cases. Six (2.8%) cases had a suspected nosocomial infection (Table 2).

Eighteen (8.3%) patients presented underlying diseases, 17/216 (7.9%) were preterms (range 31–36 + 6 weeks of gestational age), and 4/216 (1.8%) patients were on chronic pharmacological treatment (Table 1).

Fifteen (6.9%) infants were asymptomatic and admitted for clinical observation due to young age during the first and second wave of the COVID-19 pandemic, whereas 201/216 (93%) infants presented at least one symptom (Table 2). The most common symptom was fever, followed by coryza, poor feeding, cough, and gastrointestinal manifestation. Eight patients (4%) presented only gastrointestinal symptoms. None had rash (Table 3).

Ninety-eight patients were tested for concomitant viral infections; nine were positive, as reported in Table 3. None of them presented severe or critical COVID-19.

Blood tests were normal in most infants. Chest X-ray was performed in only 78/201 symptomatic infants and showed pathological findings in 42 cases (53.8%) (Table 2).

The median length of hospital stay was 4 days (IQR 3–7). Seven patients were admitted for reasons unrelated to COVID-19 (three for urinary tract infections, one for retinoblastoma, one for inguinal swelling, and one for edema of the pubic area).

Twenty-two (9.7%) patients presented with moderate/severe clinical presentation, including 15 (6.9%) with pneumonia and 7 (3.2%) with SARI (Table 3). Two infants presented initial features of Multisystem inflammatory Syndrome but did not fully meet MIS-C criteria [18]. Ten (4.6%) infants required respiratory support, and only one patient needed HFNC oxygen therapy and invasive ventilation. Five patients (2.3%) were admitted to the ICU. All patients recovered, but three presented sequelae (one coronary artery dilatation, one Horner syndrome, and one reduced motility of the right arm). Excluding paracetamol and rehydration, 33 (15.3%) infants received treatment, including antibiotics and corticosteroids (Table 3). A 78-day-old infant was treated with hydroxychloroquine in March 2020, whereas no patients received remdesivir, heparin, immunoglobulin, or immunomodulatory drugs.

According to the severity score described above, almost 90% of patients were classified as 0 and none as 4 (Table 4).

### 3.2. Correlation between Different Severity Scores and Our Clinical Outcomes

Overall, infants presented with severe/critical disease were 39/216 (18%) according to Venturini, 13/216 (6%) according to Dong, 9/216 (4.2%) according to Kanburoglu, and 64/216 (29.6%) according to Gale severity classification (Table 5).

**Figure 1:**
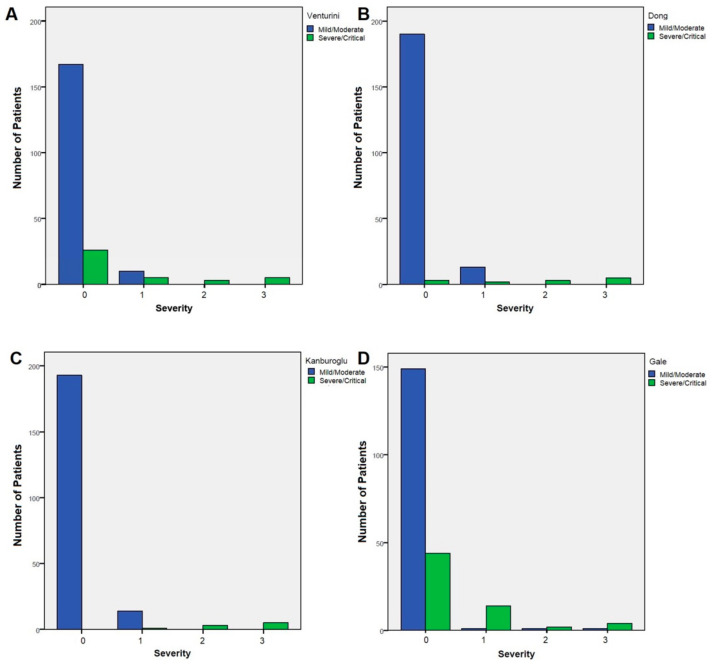
Distribution of patients classified as mild/moderate (blue bars) vs severe/critical (green bars) according to the Venturini (**A**) Dong (**B**) Kanburoglu (**C**), and Gale (**D**) severity scores among the a posteriori severity score assigned to all 216 enrolled subjects based on their clinical presentation and outcomes.

To better evaluate the accuracy of the different classifications included in the study, we performed a correlation analysis between the Dong, Kanburoglu, Gale, and Venturini classifications and the severity score assigned to all enrolled subjects according to clinical presentation. We observed a different strength of correlation between Gale (weak correlation, R = 0.355, *p* < 0.001), Venturini (moderate correlation, R = 0.425, *p* < 0.001), Dong (strong correlation, R = 0.734, *p* < 0.001), and Kanburoglu classification (very strong correlation, R = 0.859, *p* < 0.001) and the severity score used in the present study. Sensibility was 100% for all the scoring systems, while specificity increased from Gale classification (71.6%) to Venturini (83.9%), Dong (96.2%), and Kanburoglu (98.1%, Figure 1).

**Figure 2:**
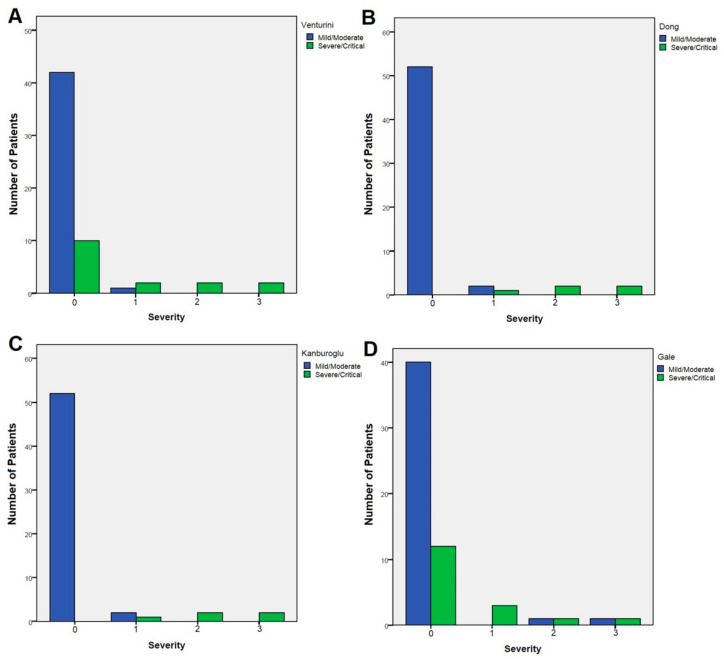
Distribution of patients classified as mild/moderate (blue bars) vs severe/critical (green bars) according to the Venturini (**A**) Dong (**B**) Kanburoglu (**C**), and Gale (**D**) severity classifications among the a posteriori severity score assigned to 59 subjects under 28 days of age based on their clinical presentation and outcomes.

A subanalysis was conducted considering only neonates (aged < 28 days), which confirmed the different performances of the scoring systems. Gale classification displayed a non-significant correlation with our clinical severity score (R = 0.239, *p* = 0.068), while Venturini correlated moderately (R = 0.485, *p* < 0.001), and both Dong (R = 0.905, *p* < 0.001) and Kanburoglu correlated very strongly (R = 0.905, *p* < 0.001) (Figure 2).

## 4. Discussion

This study is one of the most extensive series of infants less than 90 days of age admitted to Pediatric Italian Hospitals with laboratory-confirmed SARS-CoV-2 infection.

Our results suggest that the prognosis of COVID-19 in young children aligns with current published reports, with mild symptoms in most of the infected infants (48.1%), similar to older children [19,20]. Furthermore, these results are similar to a recent review and meta-analysis of more than 1200 children aged less than 5 years by Mejbah Uddin Bhuiyan et al., where half of the cases were aged less than 1 year, and a large proportion of the younger children were asymptomatic or had mild symptoms [21].

According to similar reports from other newborn and infant series [13,21,22], fever was the most reported sign (80%); rhinorrhea (27%) and cough (19%) were also described. Unlike adults, infants were more likely to present gastrointestinal symptoms such as diarrhea (13%) and vomiting (7%), as reported in older children.

Moderate to severe symptoms, such as poor feeding (24%), lethargy (4%), and respiratory distress (3%), were less frequently reported in line with current literature [23,24].

Gale reported that perinatal infection in the first 7 days after birth from a mother with SARS-CoV-2 infection is rare despite a national policy promoting keeping mother and neonate together [11]. In our cohort, 9/59 babies developed the infection in the first 7 days (early-onset neonatal COVID-19), and 50/59 between 7 and 28 days after birth (late-onset neonatal COVID-19). Whereas early-onset neonatal COVID-19 is likely related to congenital/intrapartum and perinatal transmission occurring within the first 3 days of life [25], late-onset neonatal COVID-19 is mainly associated with neonatal exposure to maternal respiratory secretions, household contacts, or, in rare cases, to infected healthcare workers during the first 28 days after birth. In line with this observation, and as already reported [20], we identified a familial history of positive contact in 58.8% of the cases, as children are likely to develop COVID-19 due to household transmission when a family member is positive for SARS-CoV-2. In our cohort, few nosocomial cases were present (2.8%), underlining the need for adequate personal protective equipment and virologic tests to assess healthcare workers’ infection status as a critical element in SARS-CoV-2 infection prevention and controls.

A previous review [19] showed that leukocytosis, lymphopenia, thrombocytopenia, and elevated inflammatory markers were the main laboratory evidence of COVID-19 infection in infants. In our cohort, instead, full blood cell count was normal in most patients, with only 9.7% of leukocytosis and 4.6% of lymphopenia. This result seems to be in contrast with adult data, where lymphocytopenia has been noted in up to 80% of critically ill subjects [2]. The limited number of severe clinical COVID-19 diseases may partly explain the low number of lymphopenias in children. Moreover, our results suggested that inflammatory markers may be abnormal only in a few patients: CRP was increased by 15.7%, ferritin by 13.9%, and LDH by 16.2%. Other reported laboratory findings were a high level of CPK (2.8%) and liver enzymes (8.8%), even if in our cohort, they were less altered than in other authors’ reports [26]. Lastly, an increased troponin level was detected in 9.7% of cases.

Chest X-rays were performed in 36.1% of infants and were abnormal in half of them. As for adults, the most frequent radiological findings were nonspecific peri-bronchial opacities and parenchymal and interstitial patterns.

Management remains supportive for neonates infected with COVID-19 and includes supplemental oxygen, respiratory support, fluid infusion, and temperature control. Currently, evidence for using antiviral medications and steroids in neonatal COVID-19 is lacking. Appropriate respiratory support, such as CPAP, is recommended in case of respiratory distress. Endotracheal intubation is more likely indicated for neonate-specific lung diseases (such as surfactant deficiency and meconium aspiration syndrome) rather than COVID-19 [26].

In our cohort, symptomatic treatment alone was used in most cases. Except for antipyretics, 33/216 patients received drugs, with most patients (28/33) receiving antibiotics, both for mild and severe cases. Mejbah Uddin Bhuiyan et al. [21] already reported that 71% of children younger than 5 years were treated with antibiotics despite having a confirmed diagnosis of COVID-19. No patients were treated with convalescent plasma or remdesivir, and only one infant received hydroxychloroquine during the first COVID-19 wave. Only 4.6% of all patients required respiratory support, and in one late preterm 19-day-old newborn, invasive ventilation was adopted.

Admissions to the hospital were mainly due to young age. When patients were stratified by different age groups, newborns were not at higher risk of severe and critical infection compared with others (39/157 (24.8%) infants vs 16/59 (27.1%) neonates). However, some previous studies have reported that neonates would have a more severe illness (in 12% of infected neonates) than older children (3% of older children required intensive care unit care) [6,7,8,9].

Interestingly, the percentage of severe/critical infants and neonates varied according to the different classifications adopted in other studies. Using the classification by Dong and Kanburoglu, we found similar results regarding the percentage of infants with severe COVID-19. Conversely, Venturini and Gale’s classifications seem to have less specificity and a higher possibility of overestimating severity in this population. Indeed, both the Gale and Venturini COVID-19 severity scores have been included in clinical parameters and laboratory findings that could frequently be abnormal in infants with respiratory tract infection, even when not severe (such as tachypnea, poor feeding, leukopenia, elevated CRP) [7,11,15,16].

For the first time, an a posteriori scoring system on clinical, radiological features, and outcomes of SARS-CoV-2 infection was applied in order to identify which of the four scores reported in the literature could better predict the objective severity of COVID-19 in neonates.

Our results showed that the clinical severity of COVID-19 in newborns and infants could vary according to the classifications used.

This finding remarks on the ambiguous definition and classification of COVID-19 in young children and points out the need for a unique, valid a priori score system for the different pediatric age groups.

This study had several limitations. First, the limited number of enrolled severe COVID-19 cases could have influenced the statistical power of our correlation analysis between severity scores. Our results highlight the needing for a unique scoring system for COVID-19 in the pediatric population. However, further studies are needed to validate our findings due to the small sample size. In addition, we could not evaluate any possible permanent long-term sequelae in infected infants by collecting data regarding only the acute phase of infection. Lastly, our cohort did not include data related to the new B.1.1.529 (Omicron) variant of concern (VOC), which is now the dominant SARS-CoV-2 variant worldwide. During the Omicron wave, we faced a significant increase in pediatric COVID-19 cases. Therefore, our results could underestimate the real burden of COVID-19 in infants; however, preliminary data have reported a low disease severity rate and omicron-related hospitalizations in adults and children compared to previous variants [27,28].

In light of this, further studies are needed to confirm the risk of COVID-19 severity in neonates and infants during the current Omicron era.

## 5. Conclusions

In conclusion, this study confirms that severe clinical manifestations are rare in neonates and young infants infected by SARS-CoV-2. However, some of them might require sustained or intensive hospital care, suggesting the need for a unique validated COVID-19 severity scoring system for neonates and infants. Furthermore, the percentage of infants with severe COVID-19 varies widely according to different authors. Therefore, further studies should be conducted to confirm our findings and design a unique clinical score valid worldwide for neonates and infants with COVID-19.

## Figures and Tables

**Table 1:** Characteristics of different scores.

	Dong et al. [7]	Gale et al. [11]	Venturini et al. [15]	Kanburoglu et al. [16]
Mild	Symptoms of acute upper respiratory tract infection or digestive symptoms		Fever and/or fatigue and/or upper airways symptoms without radiological/ultrasound findings	Upper respiratory tract infection or fever, no feeding difficulty and no obvious hypoxemia, and no risk of late neonatal sepsis
Moderate	Pneumonia without hypoxemia and shortness of breath Chest computed tomography abnormalities without clinical signs and symptoms		Fever and/or fatigue and/or upper airways symptoms (cough or mild respiratory distress) and/or poor feeding and/or pneumonia identified with chest X-ray or ultrasound	Hospitalization due to feeding difficulty or risk of late neonatal sepsis, but no obvious hypoxemia or no need for nasal continuous positive airway pressure
Severe	Oxygen saturation is <92% with other hypoxia manifestations	At least two of the following: (1) any of hyperthermia, apnea, cough, tachypnoea, respiratory distress or recession, supplemental oxygen requirement, poor feeding or vomiting, or diarrhea; (2) any of low white blood cell count, low lymphocyte count, or raised C-reactive protein concentration; and (3) abnormal chest X-ray	Fever and cough, plus at least one of the following: (1) oxygen saturation on finger pulse <92% on room air; (2) severe respiratory distress, cyanosis, intermittent apnea; (3) fast breathing; (4) systemic symptoms: drowsiness, lethargy, seizures, dehydration	Oxygen saturation <92% or need for nasal continuous positive airway pressure
Critical	Acute respiratory distress syndrome, respiratory failure, shock, encephalopathy, myocardial injury or heart failure, coagulation dysfunction, and acute kidney injury		Pediatric acute respiratory distress syndrome, sepsis-associated organ dysfunction, septic shock, coma	Mechanical ventilation or disseminated intravascular coagulopathy or multiple organ dysfunction

**Table 2:** Patients’ characteristics.

Characteristics	Number (%)
Age at diagnosis	
<2 days	3 (1.4)
2–7 days	6 (2.8)
8–14 days	11 (5.1)
15–21 days	21 (9.7)
22–28 days	18 (8.3)
>28 days	157 (72.7)
Underlying disease	18 (8.3)
Congenital heart disease	10 (6.1)
Congenital hypothyroidism	3 (1.4)
Other *	5 (2.3)
Preterms	17 (7.9)
<32 weeks	1 (0.5)
32–33 weeks	1 (0.5)
34–36	15 (6.9)
Underlying treatments	4 (1.8)
Carnitine	1 (0.5)
Levothyroxine	3 (1.4)
Known SARS-CoV-2 contact	138 (63.9)
Immediate family member	127 (58.8)
Healthcare workers	6 (2.8)
Other	5 (2.3)

* Visceromegaly (1), Neonatal jaundice (1), Neonatal Lupus (1), Metabolic disorder (1), Intraventricular hemorrhage (1).

**Table 3:** Clinical characteristics.

Characteristics	Number (%)
Clinical characteristics	
Fever	172 (79.6)
Coryza	58 (26.9)
Poor feeding	51 (23.6)
Cough	42 (19.4)
Diarrhea	28 (13.0)
Vomiting	15 (6.9)
Irritability	10 (4.6)
Lethargy	9 (4.2)
Respiratory distress	7 (3.2)
Pharyngitis	6 (2.8)
Conjunctivitis	5 (2.3)
Apnea	4 (1.8)
Febrile seizures	2 (0.9)
Asymptomatic	15 (6.9)
Laboratory findings (data available)	
Leukocytosis (210)	21 (9.7)
Lymphocytopenia (210)	10 (4.6)
Increased ESR (127)	3 (1.4)
Increased CRP (210)	34 (15.7)
Increased ALT (167)	19 (8.8)
Increased LDH (141)	35 (16.2)
Increased ferritin (141)	30 (13.9)
Increased CK (134)	6 (2.8)
Increased Troponin (124)	21 (9.7)
Concomitant viral infection (98 tested)	9 (9.2)
Adenovirus	1 (1.0)
Rhinovirus	4 (4.1)
Bocavirus	1 (1.0)
RSV	3 (3.1)
Influenza	1 (1.0)
Enterovirus	2 (2.0)
Chest X-ray performed	78 (36.1)
Normal	36 (46.2)
Parenchymal opacity	8 (10.3)
Interstitial pattern	20 (25.6)
Parenchymal and interstitial pattern	14 (17.9)

**Table 4:** Treatment and outcomes.

Characteristics	Number (%)
Moderate/severe clinical presentation	22 (10.2)
Pneumonia	15 (6.9)
SARI	7 (3.2)
Sepsis	4 (1.8)
Multisystem inflammatory syndrome	2 (0.9)
Myocarditis	1 (0.5)
Coma	1 (0.5)
Horner Syndrome	1 (0.5)
Respiratory support	10 (4.6)
Supplemental oxygen	8 (3.7)
HFNC oxygen therapy	1 (0.5)
Invasive ventilation	1 (0.5)
Treatment	33 (15.3)
Hydroxychloroquine	1 (0.5)
Macrolides	8 (3.7)
Other antibiotics	20 (9.3)
Corticosteroids	4 (1.9)
Severity score	
0	193 (89.3)
1	15 (6.9)
2	3 (1.4)
3	5 (2.3)
4	0 (0.0)
ICU admission	5 (2.3)
Sequelae	3 (1.4)
Death	0 (0.0)

**Table 5:** Severity classifications according to different definitions.

	Dong et al. [7] % (n)	Kanburoglu et al. [11] % (n)	Gale et al. [15] % (n)	Venturini et al. [16] % (n)	*p*
All Infants (n 216) Severe Critical					
	5.1% (11)	3.7% (8)	29.6% (64)	17.1% (37)	*p* < 0.001
	0.9% (2)	0.5% (1)		0.5% (2)	*p*= 0.764
Newborns (n 59) Severe					
	6.8% (4)	6.8% (4)	28.8% (17)	25.4% (15)	*p* < 0.001
Critical	1.7% (1)	1.7% (1)		1.7% (1)	*p* = 1

## Data Availability

The clinical documents related to the enrolled subjects are available from the corresponding author on reasonable request.

## References

[1] WHO Coronavirus (COVID-19) Dashboard, Updated to 10 June 2022. https://covid19.who.int/.

[2] Huang C., Wang Y., Li X., Ren L., Zhao J., Hu Y., Zhang L., Fan G., Xu J., Gu X. (2020). Clinical features of patients infected with 2019 novel coronavirus in Wuhan, China. Lancet.

[3] Guan W.J., Ni Z.Y., Hu Y., Liang W.H., Qu C.Q., He J.X., Liu L., Shan H., Lei C.L., Hui D.S.C. (2020). Clinical Characteristics of coronavirus disease 2019 in China. N. Engl. J. Med..

[4] Hernández J.L.J., Orozco I.F. (2021). COVID-19 in Children: Respiratory Involvement and Some Differences With the Adults. Front. Pediatr..

[5] Girona-Alarcon M., Bobillo-Perez S., Sole-Ribalta A., Hernandez L., Guitart C., Suarez R., Balaguer M., Cambra F.-J., Jordan I., Platform K.C. (2021). The different manifestations of COVID-19 in adults and children: A cohort study in an intensive care unit. BMC Infect. Dis..

[6] Ludvigsson J.F. (2020). Systematic review of COVID-19 in children shows milder cases and a better prognosis than adults. Acta Paediatr..

[7] Dong Y., Mo X., Hu Y., Qi X., Jiang F., Jiang Z., Tong S. (2020). Epidemiology of COVID-19 Among Children in China. Pediatrics.

[8] Lu X., Zhang L., Du H., Zhang J., Li Y.Y., Qu J., Zhang W., Wang Y., Bao S., Li Y. (2020). SARS-CoV-2 Infection in Children. N. Engl. J. Med..

[9] Lu X., Xing Y., Wong G.W.-K. (2020). COVID-19: Lessons to date from China. Arch. Dis. Child..

[10] Zimmermann P., Curtis N. (2021). Why is COVID-19 less severe in children? A review of the proposed mechanisms underlying the age-related difference in severity of SARS-CoV-2 infections. Arch. Dis. Child..

[11] Gale C., A Quigley M., Placzek A., Knight M., Ladhani S., Draper E.S., Sharkey D., Doherty C., Mactier H., Kurinczuk J.J. (2021). Characteristics and outcomes of neonatal SARS-CoV-2 infection in the UK: A prospective national cohort study using active surveillance. Lancet Child Adolesc. Health.

[12] Walker K.F., O’Donoghue K., Grace N., Dorling J., Comeau J.L., Li W., Thornton J.G. (2020). Maternal transmission of SARS-COV-2 to the neonate, and possible routes for such transmission: A systematic review and critical analysis. BJOG: Int. J. Obstet. Gynaecol..

[13] Raschetti R., Vivanti A.J., Vauloup-Fellous C., Loi B., Benachi A., De Luca D. (2020). Synthesis and systematic review of reported neonatal SARS-CoV-2 infections. Nat. Commun..

[14] Götzinger F., Santiago-Garcia B., Fumadó-Pérez V., Brinkmann F., Tebruegge M. (2021). The ability of the neonatal immune response to handle SARS-CoV-2 infection. Lancet Child Adolesc. Health.

[15] Venturini E., Montagnani C., Garazzino S., Donà D., Pierantoni L., Vecchio A.L., Nicolini G., Bianchini S., Krzysztofiak A., Galli L. (2020). Treatment of children with COVID-19: Position paper of the Italian Society of Pediatric Infectious Disease. Ital. J. Pediatr..

[16] Kanburoglu M.K., Tayman C., Oncel M.Y., Akin I.M., Can E., Demir N., Arayici S., Baser D.O., Caner I., Memisoglu A. (2020). A Multicentered Study on Epidemiologic and Clinical Characteristics of 37 Neonates With Community-acquired COVID-19. Pediatr. Infect. Dis. J..

[17] Garazzino S., Vecchio A.L., Pierantoni L., Carducci F.I.C., Marchetti F., Meini A., Castagnola E., Vergine G., Donà D., Bosis S. (2021). Epidemiology, Clinical Features and Prognostic Factors of Pediatric SARS-CoV-2 Infection: Results From an Italian Multicenter Study. Front. Pediatr..

[18] Centers for Disease Control and Prevention (2020). Emergency Preparedness and Response: Multisystem Inflammatory Syndrome in Children (MIS-C) Associated with Coronavirus Disease 2019 (COVID-19). https://emergency.cdc.gov/han/2020/han00432.asp.

[19] Dhir S.K., Kumar J., Meena J., Kumar P. (2020). Clinical features and outcome of SARS-CoV-2 infection in neonates: A systematic review. J. Trop. Pediatr..

[20] Liguoro I., Pilotto C., Bonanni M., Ferrari M.E., Pusiol A., Nocerino A., Vidal E., Cogo P. (2020). SARS-COV-2 infection in children and newborns: A systematic review. Eur. J. Pediatr..

[21] Bhuiyan M.U., Stiboy E., Hassan M.Z., Chan M., Islam M.S., Haider N., Jaffe A., Homaira N. (2021). Epidemiology of COVID-19 infection in young children under five years: A systematic review and meta-analysis. Vaccine.

[22] Mithal L.B., Machut K.Z., Muller W.J., Kociolek L.K. (2020). SARS-CoV-2 Infection in Infants Less than 90 Days Old. J. Pediatr..

[23] Olivini N., Carducci F.I.C., Santilli V., De Ioris M.A., Scarselli A., Alario D., Geremia C., Lombardi M.H., Marabotto C., Mariani R. (2020). A neonatal cluster of novel coronavirus disease 2019: Clinical management and considerations. Ital. J. Pediatr..

[24] Sankaran D., Nakra N., Cheema R., Blumberg D., Lakshminrusimha S. (2021). Perinatal SARS-CoV-2 Infection and Neonatal COVID-19: A 2021 Update. NeoReviews.

[25] Shah P.S., Diambomba Y., Acharya G., Morris S.K., Bitnun A. (2020). Classification system and case definition for SARS-CoV-2 infection in pregnant women, fetuses, and neonates. Acta Obstet. Gynecol. Scand..

[26] De Luca D. (2020). Managing neonates with respiratory failure due to SARS-CoV-2. Lancet Child Adolesc. Health.

[27] Wolter N., Jassat W., Walaza S., Welch R., Moultrie H., Groome M., Amoako D.G., Everatt J., Bhiman J.N., Scheepers C. (2022). Early assessment of the clinical severity of the SARS-CoV-2 omicron variant in South Africa: A data linkage study. Lancet.

[28] Tagarro A., Coya O.-N., Pérez-Villena A., Iglesias B., Navas A., Aguilera-Alonso D., Moraleda C. (2022). Features of COVID-19 in Children During the Omicron Wave Compared With Previous Waves in Madrid, Spain. Pediatr. Infect. Dis. J..

